# Involvement of Polyamine Metabolism in the Response of *Medicago truncatula* Genotypes to Salt Stress

**DOI:** 10.3390/plants10020269

**Published:** 2021-01-30

**Authors:** Chrystalla Antoniou, Xavier Zarza, Gholamreza Gohari, Sima Panahirad, Panagiota Filippou, Antonio F. Tiburcio, Vasileios Fotopoulos

**Affiliations:** 1 Department of Agricultural Sciences, Biotechnology and Food Science, Cyprus University of Technology Limassol, Limassol 3036, Cyprus; chrystalla.antoniou@cut.ac.cy (C.A.); giotafilippou@gmail.com (P.F.); 2 Polyamine’s Laboratory, Department of Biology, Healthcare and Environment, Faculty of Pharmacy and Food Sciences, University of Barcelona, 08028 Barcelona, Spain; xavierzarza@gmail.com (X.Z.); afernandez@ub.edu (A.F.T.); 3 Department of Horticultural Sciences, Faculty of Agriculture, University of Maragheh, 83111-55181 Maragheh, Iran; gohari.gh@maragheh.ac.ir; 4 Department of Horticultural Sciences, Faculty of Agriculture, University of Tabriz, 51666-16471 Tabriz, Iran; s.panahirad@tabrizu.ac.ir

**Keywords:** legumes, abiotic stress, polyamines, gene expression

## Abstract

Salinity constitutes one of the most important causes leading to severe reduction in plant yield. Several reports correlate the accumulation of polyamines in plants with tolerance to abiotic stress cues. The present study examined three *Medicago truncatula* genotypes with differing sensitivities to salinity (TN1.11, tolerant; Jemalong A17, moderately sensitive; TN6.18, sensitive), with the aim of examining the genotype-specific involvement of the polyamine metabolic pathway in plant response to salinity. The study was carried out with leaves harvested 48 h after watering plants with 200 mM NaCl. A comprehensive profile of free polyamines was determined using high performance liquid chromatography. All genotypes showed spermidine and spermine as the most abundant polyamines under control conditions. In salinity conditions, spermine levels increased at the expense of putrescine and spermidine, indicating a drift of polyamine metabolism towards the synthesis of increasing polycationic forms as a stress response. The increasing balance between high and low polycationic forms was clearly diminished in the salt-sensitive genotype TN6.18, showing a clear correlation with its sensitive phenotype. The polyamine metabolic profile was then supported by molecular evidence through the examination of polyamine metabolism transcript levels by RT-qPCR. General suppression of genes that are involved upstream in the PA biosynthetic pathway was determined. Contrarily, an induction in the expression of genes involved in the biosynthesis of spermine and spermidine was observed, in agreement with the metabolic analysis. A significant induction in diamino oxidase expression, involved in the catabolism of putrescine, was specifically found in the sensitive genotype ΤΝ6.18, indicating a distinct metabolic response to stress. Present findings highlight the involvement of polyamines in the defense response of *Medicago* genotypes showing sensitivity to salt stress.

## 1. Introduction

The world population is expected to increase by up to 2.3 billion by 2050, forcing agriculture and food producers to increase their production by 70% [1]. Environmental stresses are probably the biggest challenge faced by modern agriculture regarding the maintenance of plant yield [2]. Salinity stress, as one of the most frequently occurring abiotic stress factors, limits crop productivity and threatens food security. In fact, salinity causes complex phenotypic and physiologic disorders in plants by imposing ion toxicity (Na^+^ and Cl^−^) and osmotic stress, demonstrated by a significant decrease in plant yield [3].

Plants adapt to survive under various abiotic stress situations (e.g., salinity) through diverse mechanisms. The biosynthetic turnover and eventual accumulation of some compounds such as polyamines (PAs) are considered an important stress tolerance mechanism in plants [4,5]. Polyamines are naturally occurring low-molecular-weight molecules; spermidine (Spd, a triamine), spermine (Spm, a tetramine) and putrescine (Put, a diamine and mandatory precursor of Spd and Spm) being the most commonly distributed forms in plants. Polyamines are positively charged at physiological pH, and its polycationic nature is one of the important properties effectuating their biological activities. Polyamine metabolism is a complex, dynamic, and tightly regulated process involving biosynthesis, catabolism, subcellular compartmentalization, transport, and inactivation processes. Regarding the latter, PAs exist in two forms: free and conjugated. The free forms, commonly referred to as the biologically active form or cationic, are known as soluble polyamines (S-PAs). The conjugated forms, on the other hand, bind to either low-molecular-mass compounds (SH-PAs, acid-soluble polyamines) or macromolecules (PH-PAs, acid insoluble polyamines), thus reducing their net charge and activity [6]. Bas well as their important role in stress, PAs are also known to be key regulators in plant growth and development. These roles cover fundamental cellular processes (e.g., cell division, differentiation, gene expression, and DNA and protein synthesis); seed germination; root, shoot, flower and fruit development; and senescence [7]. Since the first observations from Richards and Coleman [8] regarding Put accumulation in response to potassium deficiency, several reports suggested a direct correlation between PA biosynthetic enzymes activities and PA accumulation in plant tissues [4,9]. More recently, the application of exogenous polyamines and the use of genetic engineering to increase endogenous polyamine levels in plant tissues by (*i*.) suppressing its catabolism or (*ii*.) overexpressing PA biosynthetic genes (*ADC/ODC/SAMDC/SPDS*) has been shown to enhance plant tolerance to abiotic stresses [4,10,11]. Consequently, polyamines constitute an important plant stress marker and a potential stress tolerance indicator, the molecular modus operandi of which is still not well understood.

The productivity of *Medicago truncatula*, one of the salt-sensitive crop species in legumes (*Fabaceae*), is negatively affected by soil salinity due to decreased nodular nitrogenase activity. Most legumes have no distinct salt tolerance mechanism, making them sensitive to this stress [12,13]. Regarding the importance of forage legume crops in agriculture and additionally considering *Medicago truncatula* as a primary model legume, the present study examined three *M*. *truncatula* genotypes with a differing degree of sensitivity to salinity in order to decipher the genotype-dependent involvement of the polyamine metabolic pathway under salt stress conditions.

## 2. Results

### 2.1. Phenotypic Alterations Resulting from Salt Stress in Medicago Truncatula Genotypes

**Figure 1:**
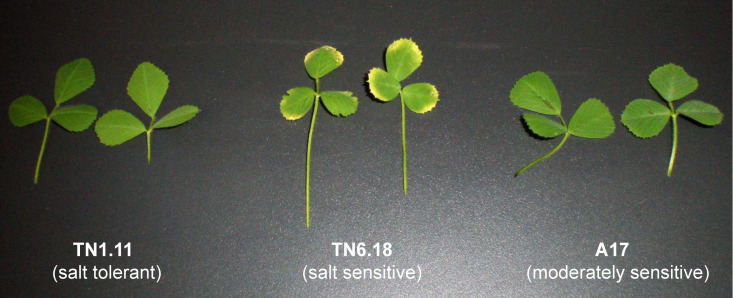
Representative image of mature leaves of TN1.11 (salt-tolerant), TN6.18 (salt-sensitive), and Jemalong A17 (moderately salt-sensitive) genotypes after 48 h 200 mM NaCl.

Phenotypic observations following 200 mM NaCl treatment for 48 h in 40 day-old plants confirmed clear salt stress-related symptoms in salt-sensitive TN6.18 genotype including chlorosis, wilting, and deformation of leaves. In contrast, the less salt-sensitive genotypes A17 and TN1.11 showed no apparent stress phenotype after salt application (Figure 1).

### 2.2. Polycationic Forms of Polyamines are Regulated in a Genotype-Specific Manner

**Figure 2:**
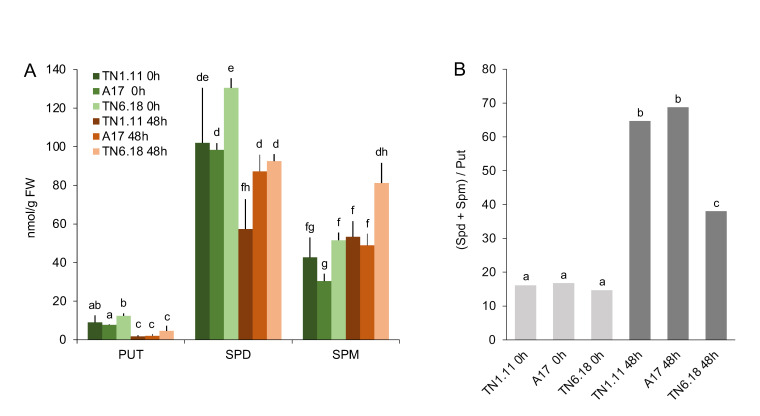
Levels of free Put (putrescine), Spd (spermidine), and Spm (spermine) (**A**) and ratio polyamines:diamine (Spd + Spm/Put) (**B**) in leaves of TN1.11, A17, and TN6.18 genotypes following 0 and 48 h salt stress (200 mM NaCl). Different tetters indicate significantly different values according to a Student–Newman–Keuls test (*P* < 0.05).

To investigate the role of polyamine metabolism in the different salt-tolerant genotypes, free polyamine content (S-PAs) was measured in the leaves. In all three genotypes, the triamine Spd was shown to be the most abundant polyamine under both control and stress conditions, followed by the tetraamine Spm and the diamine Put (Figure 2A). While Put and Spd levels showed a decreasing pattern under salt stress conditions, Spm levels showed an increasing trend in the three lines, being significant in the moderately sensitive A17 and sensitive TN6.18 genotypes, suggesting an accumulation of Spm at the expense of Put and Spd. To visualize more clearly the metabolic shift to increasing polycationic forms in response to stress, the ratio polyamine/diamine (p/d, Spd^3+^ + Spm^4+^/Put^2+^) was determined (Figure 2B). Interestingly, while all examined genotypes showed a clear increase in p/d ratio under stress conditions, the salt-sensitive TN6.18 genotype demonstrated a significantly lower p/d ratio than A17 and TN1.11 (Figure 2B). This result largely correlates with the stress phenotype observed in the different genotypes (Figure 1).

### 2.3. Transcript Levels Highlight a Distinct Regulation in the Sensitive Medicago Genotype in Response to Salt Stress Conditions

**Figure 3:**
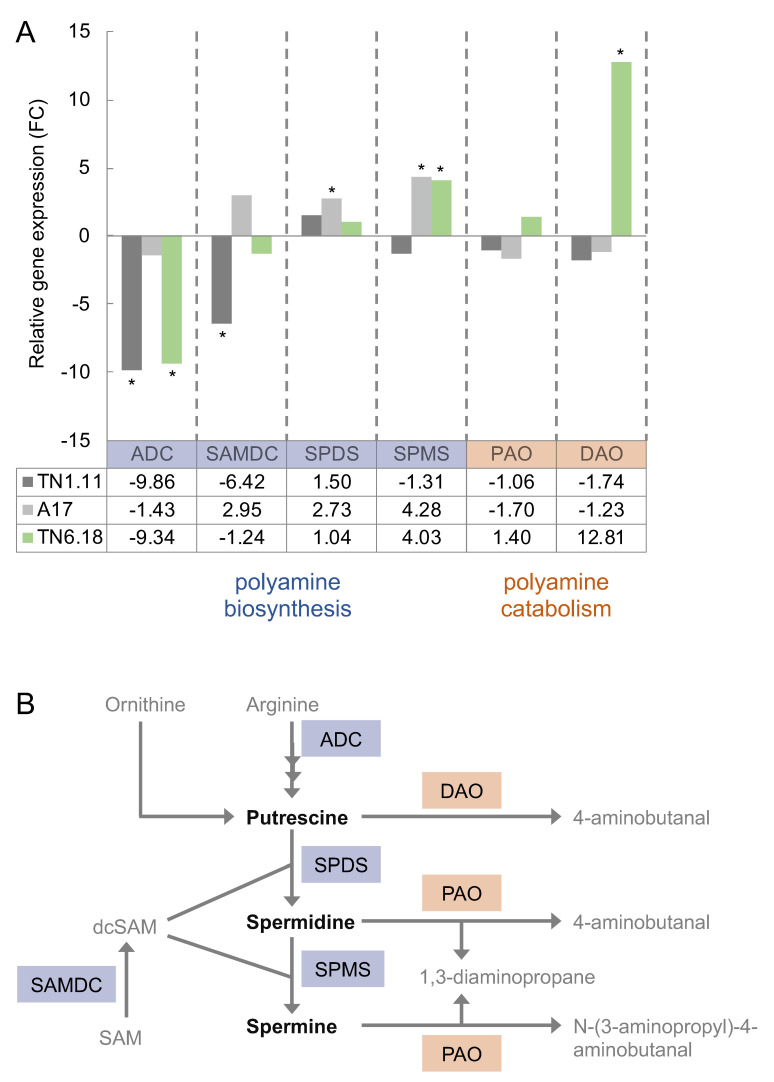
(**A**) Relative expression in fold change (FC) of genes involved in polyamine biosynthesis (*ADC*, *SAMDC*, *SPMS*, *SPDS*) and catabolism (*DAO*, *PAO*) in leaves of TN1.11, A17, and TN6.18 genotypes of *Medicago truncatula* under salt stress (200 mM NaCl) for 48 h. Results are normalized with respect to control conditions. Asterisks indicate statistically significant differences according to pairwise fixed reallocation randomization test (*p* < 0.05). (**B**) Proposed biosynthetic and catabolic pathways of polyamines (Pas) in plants. Notes: ADC, arginine decarboxylase; dcSAM, decarboxylated S-adenosylmethionine; DAO, diaminoxidase; PAO, polyaminoxidase; SAM, S-adenosylmethionine; SAMDC, S-adenosylmethionine decarboxylase; SPDS, spermidine synthase; SPMS, spermine synthase.

The metabolic differences in response to stress were further investigated by analyzing the expression of the main genes involved in both PA biosynthesis (*MtADC*, *MtSAMDC*, *MtSPMS*, and *MtSPDS*) and catabolism (*MtPAO* and *MtDAO*). As shown in Figure 3A, salinity greatly affected the gene expression levels of all transcripts examined, with the exception of *MtPAO*, which is involved in Spd and Spm catabolism (Figure 3B). In general, upstream biosynthetic genes (*MtADC*, *MtSAMDC*) appeared to be downregulated under salt stress conditions, while Spd- and especially Spm-synthase (*MtSPDS* and *MtSPMS*) were found to be significantly induced in the sensitive TN6.18 and moderately sensitive A17 genotypes. Interestingly, a significant induction in *MtDAO* gene expression, with a role in Put catabolism, was additionally noticed in the sensitive genotype ΤΝ6.18. This trend was not observed in the more tolerant genotypes TN1.11 or A17. In general, transcript levels were largely in agreement with the polyamine levels observed in response to salinity, and with the accumulation of the polycationic form Spm at the expense of Put and Spd (Figure 2).

## 3. Discussion

Put is the required diamine precursor for the more complex triamine Spd and tetraamine Spm, and the enzymes arginine decarboxylase (ADC), S-adenosylmethionine decarboxylase (SAMDC), spermidine synthase (SPDS), and spermine synthase (SPMS) have a key role in polyamine biosynthesis. ADC initiates the decarboxylation of the original amino acid backbone arginine, and SAMDC provides the required aminopropyl groups to the synthases to progressively enlarge the aliphatic structure of the polyamine. In that sense, PA catabolism acts as counterpart by oxidizing PAs and adjusting its cellular levels; diamine oxidase (DAO) and PA oxidase (PAO) are responsible enzymes in this regard [14]. Consequently, the fine-tuned balance between both synthesis and oxidation activities play an essential and complex physiological role under normal and stress conditions. All activities of the mentioned enzymes are transcriptionally regulated [15].

The significant contribution of free PAs in regulating abiotic stress tolerance responses has been confirmed through different physiological and molecular studies in plants [2,4]. PAs, as ROS scavengers and antioxidants, could adjust cell membrane stability under salinity stress, as reported in rice seedling [16]. A decrease in plant growth and biomass of TN6.18 under salinity stress compared with A17 and TN1.11 genotypes has been previously shown [17]. Supplementary studies reported a more efficient antioxidant defense mechanism in the TN1.11 genotype in comparison with A17 and TN6.18 in particular [18], representing an essential mechanism of stress tolerance. Although the individual polyamine species have been linked to some degree of tolerance in a wide range of abiotic stress conditions [19,20], the variation of its levels is largely affected by plant species, tissue, and stress intensity, making its interpretation and cross-comparison highly complex. In that sense, and under salinity stress in particular, contradictory results have often been reported. To add another layer of complexity to polyamine dynamics, it should be noted that the expression of genes involved in PA pathways are frequently tissue-specific and follow uncommon patterns in diverse plant species, most likely due to different regulation mechanisms [21]. Therefore, a more reliable, comparative, and consistent approach to analyzing polyamine homeostasis as a whole, based on the diamine/polyamine balance, has been proposed [22]. According to present findings, the balance between polyamine (Spd and Spm) and diamine (Put) levels was affected in response to salinity stress in all genotypes, leading to an increase in polycationic forms and reflecting a deviation of the Put pool to Spd and Spm synthesis. Zapata and colleagues [23] reported similar findings where increased (Spd + Spm)/Put ratio seemed to be a consistent and reliable parameter linked to salinity stress response and tolerance in several plant species. Consistently, in our assays, the ratio p/d was found to be significantly lower in the sensitive cultivar TN6.18. The in-depth analysis of the polyamine and gene expression levels indicated a clear differential response in TN6.18, potentially linked to its oxidative status, as shown in Filippou et al. (2021) [manuscript submitted for publication]. In that sense, Spm accumulation, in particular, is known to be linked to salt responses and provides increased cell membrane stability and reduce ROS accumulation [21,24,25,26,27]. In other words, increased Spm levels also reflect different degrees of exposure to stress in those tissues, in this case, in the photosynthetic tissue. This is in agreement with the increased salt sensitivity and the current Spm and SPMS response magnitude observed in A17 and TN6.18 genotypes, indicating a potential lack of salt-tolerance mechanisms in other tissues that may act as the first barrier to Na^+^ ions and therefore respond earlier to salt stress, such as the root tissue. Those early responsive mechanisms, potentially present in TN1.11 but lacking in the sensitive genotypes, may translate into faster transport and exposure to Na^+^ in upper tissues (such as the photosynthetic tissue) in the sensitive genotypes. This idea is supported by the fact that sensitive genotypes accumulate higher Spm levels and show increased oxidative stress in leaves, as shown in Filippou et al. (2021) [manuscript submitted for publication]. Interestingly, in PA catabolism, a remarkable induction in DAO was observed after imposing salinity in the salt-sensitive TN6.18 genotype. The induction of Put catabolism under salt stress has been previously shown to directly contribute to the accumulation of the compatible solute and ROS scavenger proline, the synthesis of which is strongly correlated to increased ROS levels [28]. Accordingly, unlike the moderately sensitive A17, our results suggest the activation of a second polyamine-related pathway in the sensitive TN6.18 in response to even higher oxidative levels in this genotype, as shown in Filippou et al. (2021) [manuscript submitted for publication]. These results, which will require further analysis, support the idea of a central role of polyamines in oxidative homeostasis in salt stress, and reflect the previously observed lower capacity of TN6.18 to control potentially damaging oxidative levels in the photosynthetic tissue, representing a key metabolic mechanism involved in the switch to a higher sensitivity degree in *Medicago*.

Numerous studies have been performed to increase knowledge of the molecular mechanisms involved in plant responses to abiotic stresses. As a result, several upstream and downstream processes have been identified as being connected with stress tolerance through protective mechanisms and metabolic networks in which PAs play a key role [4]. Current findings provide clear molecular and metabolic evidence that (*i*.) highly polycationic forms of polyamines are linked with plant sensitivity to salinity in a genotype-dependent manner in *Medicago* plants, with Spm potentially being considered as the key PA in this regard, and (*ii*.) the activation in parallel of a second mechanism involving putrescine oxidation is linked to increased sensitivity in *Medicago*.

## 4. Materials and Methods 

### 4.1. Plant Materials and Salt Stress Conditions

Mature (40 day-old) *Medicago truncatula* genotypes TN1.11 (tolerant), Jemalong A17 (moderately sensitive), and TN6.18 (sensitive) were used in the current study. Seeds were sown in sterile perlite:sand (1:3) pots and placed at 4 °C for 4 days for stratification. Plants were grown in a growth chamber at 22/16 °C day/night temperatures, at 60–70% RH, with a photosynthetic photon flux density of 100 μmol m^2^ s^−1^ and a 16/8 h photoperiod. Plants were watered three times per week until maturity. Water was added by weighting the pots to reach 80% of soil moisture, so as to ensure a uniform water status throughout plant development. Salinity was imposed by watering plants once with 80 mL of 200 mM NaCl solution (till leaching), and results were analyzed after 48 h according to Mhadhbi et al. [17]. Control plants were treated with water in the same manner. Experiments were performed in triplicate using pooled samples, each sample comprising tissues from a minimum of three independent plants.

Salinity was imposed by watering plants with the same amount of 200 mM NaCl solution, and results were analyzed after 48 h [17]. Control plants were treated with water in the same manner. Experiments were performed in triplicate using pooled samples, each sample comprising tissues from a minimum of three independent plants.

### 4.2. Analysis of Free PAs by HPLC

The levels of free Put, Spd, and Spm were determined by high-performance liquid chromatography (HPLC) separation of dansyl chloride-derivatized Pas, as described [29].

### 4.3. RNA Isolation, cDNA Synthesis and Real-Time RT-qPCR Assay

RNA from leaves was obtained using the RNeasy plant kit (Macherey–Nagel, Duren, Germany) followed by DNase digestion (RNase-free DNase set; Macherey–Nagel). cDNA was obtained from RNA (1 mg) using Primescript 1st Strand cDNA synthesis kit (Takara, Otsu, Japan). Real-time RT-qPCR analyses were performed with a Bio-Rad IQ5 (Bio-Rad, Hercules, CA, USA), in which the reaction mixture consisted of cDNA (4 mL) in reaction buffer (diluted 1:5), each primer (0.75 mM; Table S1; [12]), and master mix (Kapa SYBR Fast Bio-Rad iCycler2 qPCR master mix; Kapa Biosystems, Boston, MA, USA). The thermocycler conditions were 95 °C for 5 min, 95 °C for 30 s, annealing temperature for 30 s, 72 °C for 30 s, 80 °C for 2 s, and plate read at 78 °C, followed by 72 °C for 10 min. Primer annealing temperatures were 53, 56, and 60 °C. Relative quantification of gene expression and statistical analysis of all RT-qPCR data (pairwise fixed reallocation randomization test) were performed using REST software according to Pfaffl et al. [30]. All reactions were performed in triplicate, and *ACTIN* 11 was used as the housekeeping reference gene [31].

### 4.4. Statistical Analysis

SPSS was used for statistical analysis. For paired comparisons, Student–Newman–Keuls test at *P*-value < 0.05 was used, where different letters indicate significantly different values. Data shown represent the mean ± SD. The results obtained were confirmed by at least three independent experiments where at least three plants were used in each case.

## Supplementary Materials

The following are available online at https://www.mdpi.com/2223-7747/10/2/269/s1, Table S1: Oligonucleotides used as primers for real-time RT-qPCR.
