# Supplementary material for: Involvement of Polyamine Metabolism in the Response of Medicago truncatula Genotypes to Salt Stress
Source: Plants (Basel). 2021 Jan 30;10(2):269. doi: 10.3390/plants10020269 (PMC7912313; doi:10.3390/plants10020269)
Supplement: Supplementary file 1 [file plants-10-00269-s001.pdf]

Supplementary Material

Involvement of Polyamine Metabolism in the Response of *Medicago truncatula* Genotypes to Salt Stress

Chrystalla Antoniou [1], Xavier Zarza [2], Gholamreza Gohari [3], Sima Panahirad [4], Panagiota Filippou [1,†], Antonio F. Tiburcio [2] and Vasileios Fotopoulos [1,*]

Table 1. Oligonucleotides used as primers for real-time RT-qPCR.

Gene	Primer	Nucleotide sequence	Ta (°C)	Reference
Act11	Actin11-F<br>Actin11-R	5'-ATG CCA TCC TTC GTC TTG A-3'<br>5'-GCT GGT CC T GGC TGT CTC-3'	53	[Filippou et al., 2013]
ADC	ADC-F<br>ADC-R	5'-CTG GCC ATT TTG GTT CAA CT-3'<br>5'-ACC CAA ACG AAG CAA TTC AC-3'	56	[Filippou et al., 2013]
SPDS	SPDS-F<br>SPDS-R	5'-AAG GGA TGA GTG TGC GTA CC-3'<br>5'-TTT GGA GAC ATC GAC AAC CA-3'	56	[Filippou et al., 2013]
SPMS	SPMS-F<br>SPMS-R	5'-GCC AGT GAA GAA AAG GGT CA-3'<br>5'-AGA ACC ACC CAG AAA CAA CG-3'	60	[Filippou et al., 2013]
PAO	PAO-F<br>PAO-R	5'-TTT TGG CAG CAC ATG GAT AA-3'<br>5'-TTA TTC CAC CAG CAG GGA AC-3'	60	[Filippou et al., 2013]
DAO	DAO-F<br>DAO-R	5'-TGC AAT CCC AGA TGA AGT GA-3'<br>5'-CAG CTA GCA ATG TGC CAT GT-3'	60	[Filippou et al., 2013]
SAMDC	SAMDC-F<br>SAMDC-R	5'-CAA CGG TGG CGT AGA AAA AT-3'<br>5'-GCC TTC AAA ACC GAT AGC TG -3'	56	[Filippou et al., 2013]

References

Filippou, P.; Antoniou, C.; Fotopoulos, V. The nitric oxide donor sodium nitroprusside regulates proline and polyamine biosynthesis in *Medicago truncatula* plants. *Free Rad. Biol. Med.* **2013**, *56*, 172–183
